# Recent advances in targeted strategies for triple-negative breast cancer

**DOI:** 10.1186/s13045-023-01497-3

**Published:** 2023-08-28

**Authors:** Shuangli Zhu, Yuze Wu, Bin Song, Ming Yi, Yuheng Yan, Qi Mei, Kongming Wu

**Affiliations:** 1 Department of Oncology, Tongji Hospital of Tongji Medical College, Huazhong University of Science and Technology, Wuhan, 430030 China; 2 Cancer Center, Shanxi Bethune Hospital, Shanxi Academy of Medical Science, Tongji Shanxi Hospital, Third Hospital of Shanxi Medical University, Taiyuan, 030032 China; 3 Department of Breast Surgery, The First Affiliated Hospital, College of Medicine, Zhejiang University, Hangzhou, 310000 China; 4 Cancer Center, Tongji Hospital of Tongji Medical College, Huazhong University of Science and Technology, Wuhan, 430030 China

**Keywords:** Triple-negative breast cancer, Molecular subtype, Targeted therapy, Immunotherapy

## Abstract

Triple-negative breast cancer (TNBC), a highly aggressive subtype of breast cancer, negatively expresses estrogen receptor, progesterone receptor, and the human epidermal growth factor receptor 2 (HER2). Although chemotherapy is the main form of treatment for patients with TNBC, the effectiveness of chemotherapy for TNBC is still limited. The search for more effective therapies is urgent. Multiple targeted therapeutic strategies have emerged according to the specific molecules and signaling pathways expressed in TNBC. These include PI3K/AKT/mTOR inhibitors, epidermal growth factor receptor inhibitors, Notch inhibitors, poly ADP-ribose polymerase inhibitors, and antibody–drug conjugates. Moreover, immune checkpoint inhibitors, for example, pembrolizumab, atezolizumab, and durvalumab, are widely explored in the clinic. We summarize recent advances in targeted therapy and immunotherapy in TNBC, with the aim of serving as a reference for the development of individualized treatment of patients with TNBC in the future.

## Introduction

Based on the American Cancer Society, breast cancer (BC) has emerged as the second leading cause of cancer death in women, and the incidence of BC is increasing annually [1, 2]. According to the expression of biomarkers, including estrogen receptors, progesterone receptors, human epidermal growth factor receptor 2 (HER2), and Ki67, BC mainly consists of luminal A, luminal B, HER-2 overexpression, and triple-negative breast cancer (TNBC) subtypes [3]. TNBC is a specific subtype of BC, representing 15—20% of BC, and lacks expression of the estrogen receptor, progesterone receptor, and HER2 receptor on the cell surface [4, 5]. Analysis of gene expression profiles showed that TNBC was classified as a basal-like BC subtype [6]. Compared to other BC subtypes, TNBC commonly occurs in young women and is associated with increased malignancy and mortality [7, 8]. Approximately 45% of patients with TNBC have distant metastases in the brain or elsewhere, and median survival decreases from 13.3 months to 18 months [9]. Several reports have confirmed that up to 25% of patients with TNBC can recover. The Food and Drug Administration (FDA) has approved anti-metabolites, paclitaxel, and anthracyclines as adjuvant and neoadjuvant chemotherapy regimens for patients with TNBC [10, 11]. Conventional chemotherapy has shown some effectiveness in patients with TNBC. However, the toxicity of chemotherapy is harmful for patients and some patients still do not receive clinical benefit. Therefore, finding effective targets for accurate TNBC therapy is a challenging and important clinical problem to be solved [12–17].

Whole-genome sequencing studies demonstrated that TNBC is highly heterogeneous and has contributed to the classification of TNBC subtypes [18]. In recent years, "Fudan typing" has refined TNBC into various subtypes, shedding light on the accurate treatment of patients with TNBC [19, 20]. With the increasing development of histological research and the advance of bioinformatics analysis technology, cancer research is gradually developing towards large samples, multi-omics, and refinement. In recent years, potential therapeutic targets drawn from genomics, transcriptomics, metabolomics, and proteomics have emerged, and a considerable number of these research results have strong clinical translation value and have attracted widespread attention [21]. Therefore, it is necessary to develop appropriate therapeutic plans according to the unique and complex molecular characteristics and biological properties of the tumors in each TNBC patient.

Given the continuing advances in TNBC research, we summarize the fundamental characteristics and classification of TNBC and review the progress made in targeted therapy for TNBC in recent years.

## Molecular typing of TNBC

It is instructive to distinguish specific molecular typing for the treatment and prognosis determination of patients with BC. For example, TNBC patients are sensitive to chemotherapeutic agents but not endocrine therapy and TNBC patients are generally highly heterogeneous, tend to metastasize, and have a poor prognosis [22]. Therefore, clarifying the molecular typing of TNBC is important to guide individualized treatment and may further improve the treatment success rate [23].

Lehmann's team divided TNBC into the following subtypes by gene expression profile of tumor samples from 587 patients with TNBC, including basal-like 1 (BL1), basal-like 2 (BL2), mesenchymal-like (MES), mesenchymal/stem-like (MSL), immunomodulatory (IM), and luminal androgen receptor (LAR) [24]. However, this typing methodology is very homogeneous and no longer reflects the genomic characteristics of each tumor.

Currently, the most widely used is the TNBC molecular typing published by Prof. Shao Zhimin at Fudan University, known as "Fudan typing" [19, 25]. Shao's team divided 465 TNBC samples into four different subgroups by multi-omics sequencing. Namely, the LAR type, which signals through androgen receptor signaling, the MES type, which has an enrichment in growth factor signaling pathways, the IM type, which overexpresses the related signaling genes of immune cells and cytokine, and the BL type, which activates cell cycle and DNA repair with the help of reduced immune response genes [19]. This typology is similar to the results reported by Lehmann et al., but it is helpful for researchers to explore more effective individualized treatment strategies for patients with TNBC.

**Fig. 1:**
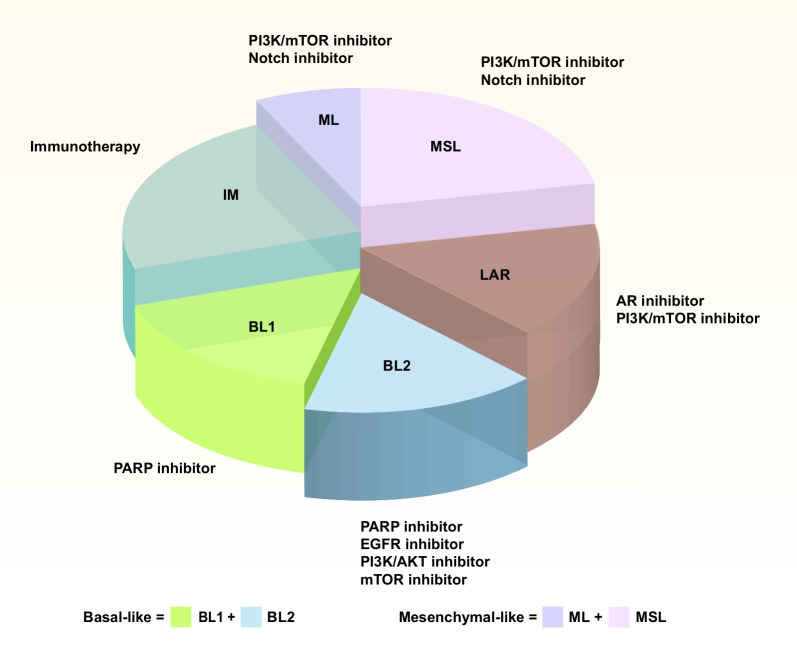
The molecular subtype of TNBC. At present, TNBC is mainly divided into the following categories, including BL1/2, IM, ML, MSL, and LAR. (The molecular subtype of TNBC was adapted from Fig. 2 in [23]) BL1: basal-like 1, IM: immunomodulatory, ML: mesenchymal-like, MSL: mesenchymal stem-like, LAR: luminal androgen receptor

In 2020, Shao's group identified androgen receptor (AR), CD8, FOXC1, and DCLK1 as immunohistochemistry (IHC) biomarkers. According to the results of IHC staining, TNBC is divided into five subtypes, including IHC-based IM (IHC-IM; AR^−^CD8^+^), IHC-based LAR (IHC-LAR; AR^+^), IHC-based basal-like immunosuppression (IHC-BLIS; AR^−^CD8^−^FOXC1^+^), immune factor-based mesenchymal (IHC-MES; AR^−^CD8^−^FOXC1^−^DCLK1^+^) and IHC-based unclassifiable (AR^−^CD8^−^FOXC1^−^DCLK1^−^). The IHC-LAR subtype demonstrates the HER2 signaling pathway activation, and the IHC-IM subtype presents an immunoinflammatory phenotype, which is characterized by the infiltration of CD8^+^ T cells into the cancer parenchyma. Moreover, the IHC-BLIS subtype exhibits a signature overexpression of vascular endothelial growth factor (VEGF). The IHC-MES subtype shows stimulation of the JAK/STAT3 (signal transducer and activator of transcription 3) signaling pathway. IHC-based subclassification offers additional information for the prognostic assessment of patients with TNBC. This makes it easier for TNBC patients to be subtyped in clinical trials and to evaluate the effectiveness of targeted therapy for selected subtypes, which would promote treating TNBC patients in a subtype-specific manner [26]. The "FUTURE typing" was first demonstrated in the FUTURE clinical trial, and the team is currently conducting a representative series of clinical trials with "FUTURESUPER", which strives to bring the treatment regime from the FUTURE study to the front line and provide more TNBC patients with new options for early individualized treatment." The development of the "FUTURESUPER" clinical trials series has greatly promoted the accurate treatment of patients with TNBC and has a broad prospective in clinical practice [27] (Fig. 1).

## TNBC-related targeted therapy

### Poly (ADP-ribose) polymerase (PARP) inhibitors

Malignant tumor cells are susceptible to the occurrence of mutations in the BRCA gene, such as the existence of mutations in the BRCA1/2 gene in patients with TNBC. BRCA1/2 plays a role in the homologous recombination repair of double-stranded DNA, and tumor cells containing mutations in the BRCA1/2 gene have defective DNA repair due to a deficiency in homologous recombination repair [28, 29]

**Fig. 2:**
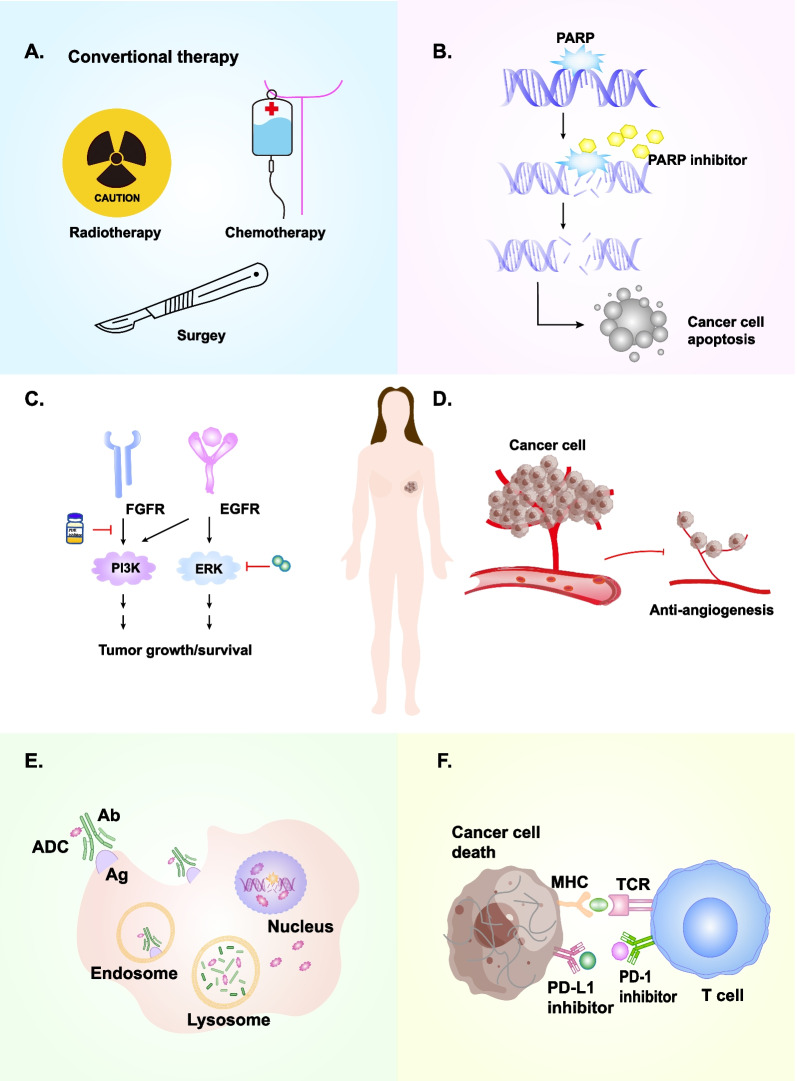
The therapeutic strategies in TNBC. There are several therapeutic strategies in TNBC. A. Traditional treatments for TNBC, including chemotherapy, radiotherapy, and surgery; B. PARP inhibitor; C. Signaling pathway-related inhibitors; D. VEGF/VEGFR inhibitors; E. ADC; F. Immune checkpoint inhibitors

PARP is a key enzyme for repairing DNA single-strand damage, and based on BRCA functional defects, PARP inhibitors are used to suppress its activity and block DNA damage repair, leading to excessive accumulation of DNA damage and ultimately to tumor cell death. Thus, PARP inhibitors could cause 'synthetic death' in BRCA1/2-deficient cancers [23, 30] (Fig. 2B). Currently, PARP inhibitors such as olaparib and talazoparib are already formally approved by the FDA for clinical therapy of patients with HER2^−^advanced or metastatic BC with BRCA mutations [24, 25] (Table 1).

**Table 1:** The clinical trials of PARP inhibitors in TNBC

Drug name	Com	Num	Trial Name	Regiment	Phase	Status	Object	POM	NCT number
Olaparib (AZD2281)		1836	OlympiA	300 mg bid po	III	Active, not recruiting	Early-stage gBRCA, adjuvant therapy	iDFS	NCT02032823
Olaparib		99		400 mg bid po (capsules) Or 300 mg bid po (tablets)	II	Completed	Advanced TNBC	ORR	NCT00679783
Olaparib		54		100 mg/400 mg bid	II	Completed	gBRCA1/2 m and advanced TNBC	ORR	NCT00494234
Olaparib		30		300 mg bid for 4 weeks of each cycle	II	Not yet recruiting	mTNBC	ORR	NCT05522491
Olaparib	Ceralasertib/ Adavosertib	273	VIOLETTE	Olaparib: 300 mg bid 28-day cycle Ceralasertib: 160 mg/d Adavosertib: 150 mg bid	II	Active, not recruiting	mTNBC	PFS	NCT03330847
Olaparib	Cediranib	155		Olaparib: 100–400 mg po bid on days 1–28 Cediranib: po	I/II	Active, not recruiting	mTNBC	PFS	NCT01116648
Olaparib	Durvalumab	45	DORA	Olaparib: 300 mg bid Durvalumab: iv every 28 days	II	Completed	Advanced TNBC	PFS	NCT03167619
Olaparib	Durvalumab (MEDI4736)	264	MEDIOLA	Olaparib: 300 mg bid for 4 weeks Durvalumab: q4w starting on day 1	I/II	Active, not recruiting	gBRCAm HER2-mBC/TNBC	PFS	NCT02734004
Olaparib	Durvalumab	3		Olaparib: po bid for 28 days Durvalumab: iv over 1 h on day 1	I	Completed	mTNBC	CLIA	NCT03544125
Olaparib	Durvalumab	132		Olaparib: po bid on days 1–28 Durvalumab: iv over 1 h on day 1	II	Recruiting	mTNBC	ORR	NCT03801369
Olaparib	Physician's choice chemotherapy	302	OlympiAD	Olaparib: 300 mg bid po Capecitabine: 2500 mg/m^2^ day 1-14 or Vinorelbine 30 mg/m^2^ day 1,8 or Eribulin 1.4 mg/m^2^ day 1,8	III	Active, not recruiting	Advanced/Metastatic gBRCA, ≤ 2 prior lines	PFS	NCT02000622
Olaparib	Paclitaxel	19		Olaparib: 200 mg bid Paclitaxel: iv over 1 h	I/II	Completed	mTNBC	AEs	NCT00707707
Olaparib	Radiation therapy	24		Olaparib: five levels of dose, po bid each day IMRT	I	Unknown	Advanced or mTNBC	MTD	NCT03109080
Veliparib (ABT-888)	Temozolomide	294		Veliparib: 40 mg bid days 1–7 Temozolomide: 150 to 200 mg/m^2^ qd days 1–5 in each 28-day cycle	II	Completed	Metastatic gBRCA, ≤ 0–2 prior lines	PFS	NCT01506609
Veliparib	Carboplatin and Paclitaxel	294		Veliparib: 80 mg bid day 1–7 Carboplatin: day 3 of each 21-day cycle Paclitaxel: 175 mg/m^2^ on day 3 of each 21-day cycle	II	Completed	Metastatic gBRCA, ≤ 0–2 prior lines	PFS	NCT01506609
Veliparib	Carboplatin and Paclitaxel	509	BROCADE3	Veliparib: 120 mg bid on day 2–5 of a 21-day cycle Carboplatin: iv AUC 6 mg/ml/min on day 1 of every cycle Paclitaxel: 80 mg/m^2^ iv on day 1, 8, and 15 of every cycle	III	Active, not recruiting	Metastatic or advanced gBRCA1/2 m HER2- BC/TNBC	mPFS	NCT02163694
Veliparib	Carboplatin and Paclitaxel	634	BrighTNess	Veliparib: 50 mg po bid Paclitaxel: 80 mg/m2 iv weekly for 12 doses Carboplatin: AUC 6 mg/mL/min iv q3w for 4 cycles	II	Completed	Stage II or III TNBC Neoadjuvant	pCR	NCT02032277
Veliparib	Carboplatin and Paclitaxel	116	I-SPY 2	NA	II	Recruiting	Stage II or III TNBC Neoadjuvant	pCR	NCT01042379
Veliparib	Cyclophosphamide	124		Veliparib: 60 mg po by mouth Cyclophosphamide: 50 mg po by mouth for 21d	II	Completed	Advanced TNBC	ORR, PFS	NCT01306032
Talazoparib (BMN 673)		431	EMBRACA	1.0 mg/d po for 21 continuous days	III	Completed	Advanced/Metastatic gBRCA, ≤ 3 prior lines	PFS	NCT01945775
Talazoparib		84	ABRAZO	1 mg qd	II	Terminated	gBRCA1/2 m advanced TNBC	ORR	NCT02034916
Talazoparib		36		1 mg/d po for 6 months	II	Completed	gBRCA1/2 m and operable HER2- BC/TNBC	pCR	NCT02282345
Niraparib		216	BRAVO	300 mg (3 × 100 mg capsules) /d po for 21 continuous days	III	Terminated	Advanced/Metastatic gBRCA, ≤ 2 prior lines	PFS	NCT01905592
Niraparib	Pembrolizumab	122	TOPACIO	Niraparib: 300 mg/d po on Day 1-21 Pembrolizumab: 200 mg iv on Day 1 of each 21-day cycle	I/II	Completed	Advanced TNBC	DLTs, ORR	NCT02657889

iDFS: invasive disease-free survival; mTNBC: metastatic triple negative breast cancer; HER2-: HER2 negative; mBC: metastatic breast cancer; gBRCA1/2 m: germline BRCA1/2 mutated; ORR: overall response rate; PFS: progression-free survival; CLIA: Clinical Laboratory Improvement Act; AEs: adverse events; IMRT: intensity modulated radiotherapy; MTD: maximum tolerated dose; DLTs: dose-limiting toxicity; Com: Combination; Num: Number; POM: Primary outcome measures

Olaparib is ineffective in metastatic TNBC (mTNBC) patients and wild type BRCA1/2, but a clinical trial revealed higher objective remission rates with olaparib monotherapy in untreated TNBC [31]. The OlympiAD trial compared the progression-free survival (PFS) of patients with HER2-negative metastatic BC who received olaparib monotherapy or standard therapy. The results indicated that compared with standard therapy, median PFS with olaparib monotherapy lasted 2.8 months longer and reduced disease progression or risk of death by 42% [32]. Analysis of follow-up results showed that overall survival (OS) was prolonged for BC patients given first-line olaparib compared to the standard group. In patients with TNBC, although the olaparib group prolonged OS, the difference was not statistically significant [33]. Interestingly, response to olaparib was correlated with low RAD51 scores, high TIL or high PD-L1 expression.

Another trial, OlympiA, evaluated the effectiveness and side effects of olaparib compared to placebo in the adjunctive therapy of patients with early-stage HER2-negative BC who carry the BRCA1/2 germline mutation. Results revealed that olaparib significantly improved patient OS compared to the placebo group, with 3-year invasive disease-free survival (iDFS) of 85.9% compared to 77.1% in the placebo group, and distant disease-free survival (DDFS) was 87.5% compared to 80.4% in the placebo group. Furthermore, olaparib had no serious adverse effects [34].

Talazoparib was taken by patients with advanced BC carrying BRCA1/2 germline mutation in the EMBRACA trial, showing notably longer PFS (8.6 months versus 5.6 months) in the talazoparib arm versus the chemotherapy arm and objective response rate (ORR) was improved (62.6% versus 27.2%) [35]. In addition, the NEOTALA trial explored the effectiveness of talazoparib alone in the neoadjuvant therapy of patients with HER2-negative BC who have BRCA1/2 germline mutations. It demonstrated significantly higher pathologic complete response (pCR) rates in the evaluable and intent-to-treat populations (all TNBC patients) (45.8% and 49.2%), respectively, with a well-tolerated safety profile [36]. Sequential combination therapy with talazoparib and carboplatin suppressed primary cancer cell growth and distant metastases in patients with TNBC, laying the foundation for treating early-stage TNBC [37].

Although PARP inhibitors are effective for TNBC, clinical resistance cannot be ignored, hence need to explore resistance mechanisms further and find better and more effective treatment strategies [38].

### AR inhibitors

The LAR type is driven via the AR signaling pathway, and the level of AR expression in the LAR is negatively correlated with PFS and OS in TNBC patients [39, 40]. Currently, researchers have explored many AR inhibitors for TNBC therapy [41] (Table 2). Although clinical trials have demonstrated that AR inhibitors have been effective in the therapy of TNBC patients, the exact mechanism is unclear.

**Table 2:** The clinical trials of AR inhibitors in TNBC

Drug name	Com	Num	Regiments	Ph	State	Object	POM	NCT number
Bicalutamide		60	150 mg/d po	II	Unknown	AR^+^TNBC	CBR, PFS	NCT02353988
Bicalutamide		36	150 mg/d po	III	Terminated	AR^+^TNBC	CBR	NCT03055312
Bicalutamide		1	150 mg/d po	II	Terminated	AR^+^TNBC	CBR	NCT02348281
Bicalutamide	Ribociclib	37	Bicalutamide: 150 mg/d po Ribociclib: 400 mg/d po	I/II	Recruiting	AR^+^TNBC	MTD, CBR	NCT03090165
Enzalutamide		50	160 mg/d po for 52 weeks	II	Active, not recruiting	AR^+^TNBC	feasibility	NCT02750358
Enzalutamide	Paclitaxel	37	Enzalutamide: PO daily on days 1–7 Paclitaxel: iv over 2 h on day 1. Treatments repeat every 7 days for up to 12 cycles	II	Recruiting	AR^+^TNBC	pCR	NCT02689427
GTx-024		32	18 mg/d po	II	Terminated	AR^+^TNBC	CBR	NCT02368691
GTx-024	Pembrolizumab	18	GTx-024: 18 mg/d po Pembrolizumab: iv over 30 min on day 1	II	Active, not recruiting	AR^+^TNBC	RR	NCT02971761

AR^+^TNBC: androgen receptor positive triple negative breast cancer; CBR: clinical benefit rate; PFS: progression-free survival; MTD: maximum tolerated dose; pCR: pathologic complete response; RR: response rate

AR^+^ expression was confirmed in approximately 12% of ER^−^PR^−^ BC patients. Patients received bicalutamide and showed a clinical benefit rate (CBR) of 19% and 3 months mPFS, and the patients were well tolerated [42]. Enzalutamide demonstrated favorable clinical effectiveness and tolerance in patients with AR^+^ TNBC, mPFS and mOS were 3.3 and 17.6 months, and serious adverse events in patients were 2%. Thus, it is recommended that enzalutamide may be used to treat patients with AR^+^ TNBC [43]. UCBG 12–1 is a trial on the effectiveness of abiraterone plus prednisolone in AR^+^ advanced TNBC patients. The results indicated that patients treated with abiraterone had an mPFS of 7.5 months, an ORR of 8.22%, a CBR in 20% of patients, and manageable adverse events [44].

Additionally, researchers performed a series of studies combining AR inhibitors with other TNBC treatment regimens. Min et al. discovered that a combination of the AR inhibitor AZD3514 and olaparib played a synergetic effect role in BC cells by modulating the DNA damage response [45]. Likewise, combining an AR inhibitor with a PARP inhibitor repressed the progression of TNBC cells [46]. The above preclinical trials suggested that AR inhibitors combined with PARP inhibitors may have favorable CBR in treating TNBC patients.

Subsequently, researchers designed clinical trials related to AR inhibitors and other agents. TBCRC032 is a multicenter clinical trial, which investigates the effectiveness of enzalutamide and taselisibin AR^+^TNBC patients. The study demonstrated that combination therapy effectively increased the CBR of patients with TNBC (35.7%), and the mPFS was 3.4 months [47]. Moreover, Choupani et al. found that enzalutamide combination with cyclin-dependent kinase (CDK) 4/6 inhibitor ribociclib had synergistic tumor-inhibiting effects on TNBC cells [48]. Although preclinical data of AR inhibitors in combination with CDK4/6 inhibitors have shown promising antitumor effects, relevant clinical trials are still ongoing, and data are not yet available (Table 2).

Currently, most studies on the AR inhibitor in treating TNBC patients are I/II clinical trials, and there is a lack of large specimen data in phase III/IV to further explore the effectiveness of AR inhibitor in TNBC patients. Likewise, it is worth exploring whether AR inhibitors combined with other drugs such as PARP inhibitors and immunotherapy will bring about better clinical effects.

### CDK inhibitors

CDK is a key enzyme that regulates transition in the various phases of the cell cycle, and continued activation can result in tumor cell proliferation [49]. DK4/6 inhibitors primarily inhibit the G1-S phase, thereby inhibiting the cellular DNA replication process [50]. The LAR subtype is highly sensitive to CDK4/6 inhibitors. Thus, using CDK4/6 inhibitors may be a potential therapeutic approach for the LAR subtype [51]. The FDA has already approved CDK4/6 inhibitors to treat TNBC patients, concluding palbociclib and ribociclib [52] (Table 3).

**Table 3:** The clinical trials of CDK inhibitors in TNBC

Drug name	Com	Num	Regiments	Ph	State	Object	POM	NCT number
Palbociclib	Binimetinib	24	Palbociclib: 100 mg/d po, 21 days on/7 days off Binimetinib: 45 mg bid po, 21 days on/7 days off	I/II	Active, not recruiting	mTNBC	PFS	NCT04494958
Palbociclib	Paclitaxel/ carboplatin	126	Palbociclib: 125 mg/d po on days 1–14 Paclitaxel: 80 mg/m^2^ iv on day 1, 8, 15 and 22 Carboplatin: AUC 2 iv on day 1, 8, 15 and 22	II	Not yet recruiting	TNBC	Early metabolic response	NCT05067530
Palbociclib	Avelumab	45	NA	I	Recruiting	AR^+^TNBC	MTD	NCT04360941
Trilaciclib	Gemcitabine/ Carboplatin	102	Trilaciclib: 240 mg/m^2^ iv on day 1, 8 Gemcitabine: 1000 mg/m^2^ on day 1, 8 Carboplatin: AUC 2 on day 1, 8 of each 21-cycle	II	Terminated	mTNBC	DSN	NCT02978716
Trilaciclib	Gemcitabine/ Carboplatin	194	Trilaciclib: 240 mg/m^2^ iv on day 1, 8 Gemcitabine: 1000 mg/m^2^ on day 1, 8 Carboplatin: AUC 2 on day 1, 8 of each 21-cycle	III	Active, not recruiting	mTNBC	OS	NCT04799249
Trilaciclib	Sacituzumab Govitecan	30	Trilaciclib: 240 mg/m^2^ iv on day 1, 8 Sacituzumab Govitecan: 10 mg/kg	II	Active, not recruiting	TNBC	PFS	NCT05113966
Trilaciclib	Doxorubicin/ Cyclophosphamide/ Pembrolizumab	24	Trilaciclib: 240 mg/m^2^ Doxorubicin: 60 mg/m^2^ q2w for the first 4 cycles Cyclophosphamide: 600 mg/m^2^ for cycles 5–16 Pembrolizumab: 400 mg iv q6w for cycles 1, 4, 9, 15	II	Completed	TNBC	pCR	NCT05112536
Trilaciclib	Epirubicin/ Cyclophosphamide/ Pembrolizumab	150	Trilaciclib: 240 mg/m^2^ iv on day 1 Epirubicin: 100 mg/m^2^ iv on day 1, q2w/q3w Cyclophosphamide:600 mg/m^2^ on day 1, q2w/q3w Paclitaxel:100 mg/m^2^ iv on day 1,8,15, q3w, 4 cycles	II	Not yet recruiting	TNBC	The incidence of CIN	NCT05862610
Etoposide	Anlotinib	100	Etoposide: 75 mg/d po on day 1-10, 21 days/cycle Anlotinib: 12 mg/d po on day 1-14, 21 days/cycle	II	Recruiting	Advanced TNBC	ORR	NCT04452370
Abemaciclib	Surgery	200	Abemaciclib: PO BID on days 1–14 or days 1–21 Surgery: no later than 12 weeks after the last dose of neoadjuvant chemotherapy	II	Recruiting	Refractory TNBC	Incidence of AEs	NCT03979508
Prexasertib		111	105 mg/m^2^ iv once every 14 days, 28 days/cycle	II	Terminated	TNBC	Objective Response	NCT02203513
Prexasertib	LY3023414	10	Prexasertib: 80 mg/m^2^ iv q2w LY3023414: 150 mg bid	II	Active, not recruiting	mTNBC	ORR	NCT04032080

mTNBC: metastatic triple negative breast cancer; PFS: progression-free survival; AUC: area under the curve; AR^+^TNBC: androgen receptor-positive triple negative breast cancer; MTD: maximum tolerated dose; DSN: duration of Severe (Grade 4) Neutropenia; OS: overall survival; pCR: pathologic complete response; ORR: overall response rate; AEs: adverse events; NA: not acquire

Several preclinical trials have shown the combination of CDK4/6 with other targeted drugs plays a favorable antitumor role in TNBC cells. Sequential combination therapy with palbociclib and paclitaxel could more effectively suppress TNBC cell proliferation [53]. Shao's team suggested that palbociclib combination with olaparib indicated synergistic antitumor effects in TNBC cells [54]. Similarly, this phenomenon is also present in other CDK and PARP inhibitors [55]. Moreover, ribociclib and PI3K inhibitor BYL719 can significantly promote G1 phase arrest in TNBC cells. Furthermore, ribociclib and BYL719 with an immune checkpoint inhibitor (ICI) resulted in complete tumor regression in TNBC xenograft models [56, 57]. Circular RNA has been related to prognosis in TNBC patients, and downregulation of circEIF3M inhibit CND1, which interacts with CDK4 to cause G1 phase arrest in TNBC cells [58].

In the PALOMA-2 trial, palbociclib and letrozole notably improved PFS in ER^+^/HER2^−^ BC patients in both the general and Asian populations [59, 60]. The PALOMA-3 trial assessed the efficacy of combination therapy with palbociclib and fulvestrant in ER^+^/HER2^−^ BC patients. ​Patients receiving palbociclib and fulvestrant extended PFS and OS compared with controls [61, 62]. Nevertheless, the PALLAS trial demonstrated that combining endocrine therapy and palbociclib failed to improve PFS compared to endocrine therapy alone in ER^+^/HER2^−^ BC patients [63–65]. Additionally, other studies have shown that palbociclib is ineffective in combination with chemotherapy [66].

Similar to palbociclib, ribociclib combined with fulvestrant significantly improved OS in ER^+^/HER2^−^ BC patients [67]. Compared to palbociclib, abemaciclib combined with endocrine therapy may prolong iDFS in patients with ER^+^/HER2^−^ BC and has favorable safety [68]. Moreover, abemaciclib combined with fulvestrant in treating ER^+^/HER2^−^ BC patients significantly improved PFS and ORR [69]. Several clinical trials related to CDK4/6 inhibitors are underway and we expect good results.

### PI3K/AKT/mTOR signaling pathway inhibitors

**Fig. 3:**
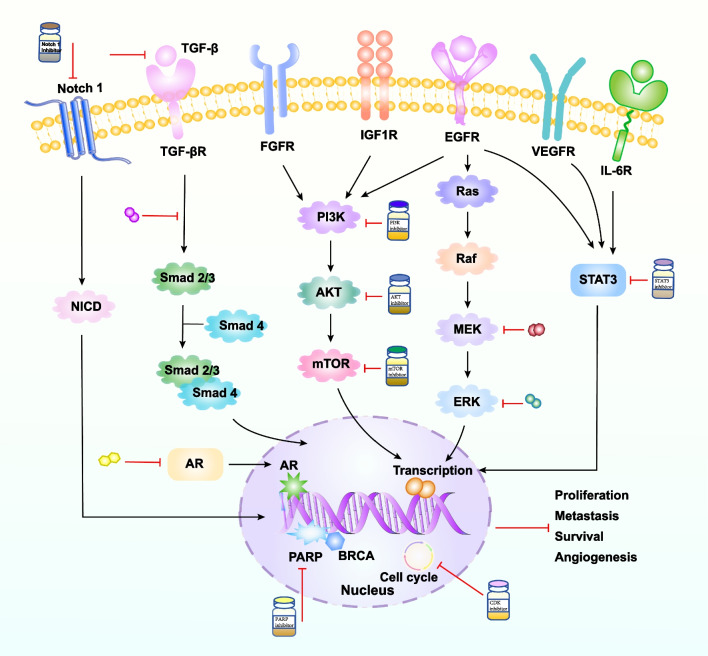
The signaling pathway and its inhibitors of TNBC. Presentation of TNBC-related signaling pathways and their inhibitors. Excitatory regulation is symbolized by black arrows, red arrows stand for inhibitory effects. (The TNBC signaling pathway and its inhibitors were adapted from Fig. 2 in [79])

The PI3K/AKT/mTOR signaling pathway, the most prevalent cancer activation pathway, results in proliferation and a host of other malignant biological behaviors in tumor cells [70, 71]. PI3K, a critical protein in this signaling pathway, mediates tumor cell growth, proliferation, and metabolism. In addition, PI3K/AKT pathway is an important intracellular signaling pathway, which participates in the expression of genes linked to proliferation and apoptosis in cancer cells. For example, activation of AKT can regulate the expression of its downstream proteins such as cyclin A1, cyclin D1, Bax, Bcl-2, and others. Thus mediate the malignant biological behavior of various tumors [72]. Multiple genomic alterations resulted in activated PI3K pathways, such as PIK3CA and AKT [73], and act as oncogenic drivers promoting tumor cell transformation, tumor initiation, progression, and apoptosis [74]. Mutations in PIK3CA lead to tumorigenesis [75, 76]. A study has shown that PIK3CA was mutated in 20% to 40% of BC and was associated with increased resistance to chemotherapy [77]. PI3KCA mutations have been reported in approximately 10% of TNBC, but are more common in the LAR and MES subtypes. Therefore, inhibiting the PI3K/AKT/mTOR signaling pathway might be a prospective approach for treating breast cancer [19, 78, 79] (Fig. 3).

Currently, relevant studies are exploring the PI3K/AKT/mTOR signaling pathway inhibitors, including capivasertib and ipatasertib. Several inhibitors have been considered in preclinical studies or clinical trials [80] (Table 4).

**Table 4:** The clinical trials of signaling pathway inhibitors

Target	Drug name	Com	Num	Regiments	Phase	Status	Object	POM	NCT number
PI3K inhibitor	Alpelisib (BYL719)	nab-paclitaxel	137	Alpelisib: 300 mg qd po nab-paclitaxel: 100 mg/m^2^ iv on Days 1, 8 and 15 of a 28-day cycle	III	Active, not recruiting	TNBC	PFS, ORR	NCT04251533
	Alpelisib (BYL719)	nab-paclitaxel	8	Alpelisib: qd po on days 1–21 Nab-paclitaxel: iv over 30 min on days 1, 8, and 15	II	Active, not recruiting	TNBC	pCR	NCT04216472
	AZD8186		147	A single dose on day 1 followed by ongoing multiple dosing	I	Completed	TNBC	Safety and tolerability	NCT01884285
	BKM120 (Buparlisib)		50	100 mg/d po in cycles of 28 days	II	Completed	TNBC	CBR	NCT01790932
	BKM120		50	100 mg/d po in cycles of 28 days, until disease progression	II	Completed	TNBC	CBR	NCT01629615
	BKM120	Capecitabine	10	BKM120: 100 mg/d po Capecitabine: 500 mg bid po, 14 days on and 7 days off	II	Completed	mBC	CBR	NCT02000882
	BKM120/BYL719	Olaparib	118	BKM120: 40 mg/d po Olaparib: 50 mg bid po BYL719: 250 mg/d po	I	Completed	TNBC	MTD	NCT01623349
	CUDC-907		43	60 mg/d po 5 days on 2 days off until disease progression	I	Completed	TNBC	Safety and tolerability	NCT02307240
	GDC-0941	Cisplatin	11	GDC-0941: 260 mg po, days 2–6, 9–13, 16–20, 23–27. 28 days cycle Cisplatin: 25 mg/m^2^ iv day 1, 8, 15	I/II	Terminated	TNBC	Safety and tolerability, ORR	NCT01918306
	SF1126		44	Dose Escalating with 3 + patients in each cohort	I	Completed	Solid cancer	DLTs	NCT00907205
Akt inhibitor	GSK2141795	Trametinib (GSK1120212)	37	GSK2141795: po qd on days 1–28 Trametinib: po qd on days 1–28	II	Completed	TNBC	ORR	NCT01964924
	GSK2141795	Trametinib	240	Po qd	I	Completed	TNBC	Safety and tolerability	NCT01138085
	GSK2141795	Trametinib	37	Po qd on days 1–28	II	Completed	TNBC	ORR	NCT01964924
	ONC201 (TIC10)		4	Po on days 3, 10, and 17	II	Terminated	TNBC	ORR	NCT03733119
	Ipatasertib (GDC-0068)	Paclitaxel	151	Ipatasertib: 400 mg/d po on days 1–21 of each 28-day cycle for 3 cycles Paclitaxel: 80 mg/m^2^ iv q1w	II	Completed	TNBC	pCR	NCT02301988
	Ipatasertib	Paclitaxel	124	Ipatasertib: 400 mg/d po days 1–21 in each cycle of 28 days Paclitaxel: 80 mg/m^2^ iv on days 1, 8, and 15	II	Completed	TNBC	PFS	NCT02162719
PI3K /mTOR inhibitor	PF-05212384 (Gedatolisib)	Docetaxel/ Cisplatin	110	90 mg/m^2^ iv as a 3 weeks cycle Docetaxel/Cisplatin: 75 mg/m^2^ iv once q3w	I	Completed	TNBC	DLTs, ORR	NCT01920061
	BEZ235	MEK162	29	NA	I	Completed	TNBC	DLTs	NCT01337765
	PQR309	Eribulin (Halaven®)	41	PQR309: after eribulin Eribulin: 1.4 mg/m^2^ iv on day 1, 8 in a period of 21 days	I	Completed	TNBC	AEs, SAEs	NCT02723877
EGFR inhibitor	Cetuximab (Erbitux)	Ixabepilone (Ixempra ®)	40	Cetuximab: 400 mg/m^2^ iv over 120 min on day 1 of the first of four 21 days cycles Ixabepilone: 40 mg/m^2^ iv over 180 min on day 1 of each of four 21 days cycles	II	Completed	TNBC	CRR	NCT01097642
	Lapatinib (Tykerb ®)	Veliparib (ABT-888)	23	Lapatinib:1250 mg/d for 28 days Veliparib: 200 mg/bid for 28 days	NA	Active, not recruiting	TNBC	Safety and toxicity	NCT02158507
	Lapatinib	Everolimus (mTOR inhibitor)	5	Lapatinib: 1250 mg by mouth daily Everolimus: 5 mg by mouth daily	II	Terminated	TNBC	Safety and toxicity, ORR	NCT01272141
	Erlotinib (Tarceva ®)	Cisplatin plus temsirolimus	9	Erlotinib: 100 mg by mouth daily Cisplatin: 30 mg/m^2^ iv weekly on days 1 and 8 of a 3 weeks cycle Temsirolimus: dosing level, 15 mg, 25 mg	I	Completed	TNBC	MTD	NCT00998036
	Erlotinib	Metformin	8	Erlotinib: 150 mg/d Metformin: 850 mg bid	I	Completed	TNBC	MTD	NCT01650506
	Erlotinib	Bendamustine	11	Erlotinib: 100 or 150 mg po on days 5—21 of each 28 days cycle Bendamustine: 100 or 120 mg/m^2^ iv on days 1 and 2	I/II	Completed	Breast Cancer	MTD, DLTs, PFS	NCT00834678
	Gefitinib (Irresa ®)		50	250 mg/d by mouth until disease progression	II	Unknown	TNBC	CBR	NCT01732276
Notch inhibitor	AL101		67	NA	II	Active, not recruiting	TNBC	ORR	NCT04461600
	RO4929097 (R4733)		6	Po qd on days 1–3, 8–10, and 15–17	II	Terminated	TNBC	ORR, PFS	NCT01151449
	RO4929097	Carboplatin plus Paclitaxel	14	RO4929097: po qd on days 1–3, 8–10, and 15–17 Paclitaxel: iv over 60 min on days 1, 8, and 15 Carboplatin: iv over 60 min on day 1	I	Terminated	TNBC	AEs, MTD	NCT01238133
TGF-β inhibitor	Bintrafusp Alfa (M7824)		11	1200 mg once q2w	II	Completed	TNBC	ORR	NCT04489940

PIK3: phosphoinositide-3-kinase; TNBC: triple negative breast cancer; PFS: progression-free survival; pCR: pathologic complete response; CBR: clinical benefit rate; MTD: maximum tolerated dose; AKT: serine/threonine kinase; mTOR: mammalian target of rapamycin; EGFR: epidermal growth factor receptor; DLT: dose-limiting toxicity; AEs: adverse events; SAEs: serious adverse events; TGF-β: transforming growth factor Beta; mTNBC: metastatic triple negative breast cancer; HER2-: HER2 negative; mBC: metastatic breast cancer; gBRCA1/2 m: germline BRCA1/2 mutated; ORR: overall response rate; CRR: complete response rate

Dey et al. summarized the PI3K inhibitors currently used in TNBC clinical trials [18]. LY294002, the first synthetic PI3K inhibitor, was used to explore the mechanism of AKT inhibitor induced-apoptosis [81]. SF1126 is a chemically modified form of LY294002, shown to inhibit tumor initiation and angiogenesis in vivo [82, 83]. A phase I trial showed that SF1126 had no dose-limiting toxicity or hepatotoxicity and showed comparable efficacy against several solid tumors [84]. However, SF1126 was a potential cancer treatment, and its target mechanism in TNBC was unclear. Deng et al. found that SF1126, in combination with gefitinib, induced apoptosis of TNBC cells by blocking the EGFR-PI3K-AKT-mTOR pathway [85]. Furthermore, SF1126 combination with sorafenib showed a favorable antitumor effect of hepatocellular carcinoma in vivo [86]. Therefore, it is important to further explore the mechanism of PI3K inhibitors targeting TNBC.

In addition to PI3K inhibitors, there are several AKT inhibitors in clinical trials [87]. AZD5363 has been administered as monotherapy to treat patients with BC, gastric and prostate cancers [88]. The AKT inhibitor ipatasertib has been used as monotherapy for TNBC patients [89]. Results from the EAY131-Y subgroup of the NCI-MATCH study showed that capivasertib had antitumor activity in a range of metastatic tumors with AKT1/E17K mutations [90]. The LOTUS and PAKT studies showed that adding the AKT inhibitors ipatasertib or capivasertib to first-line paclitaxel therapy in mTNBC prolonged PFS in patients, with more apparent CBR in patients carrying PIK3CA/AKT1/PTEN mutations [91, 92]. The effectiveness of neoadjuvant ipatasertib combined with paclitaxel in early TNBC was also evaluated in the FAIRLANE study. The result demonstrated that the pCR was higher in the ipatasertib arm than in the placebo arm in patients with mutations in the PI3K/AKT/mTOR signaling pathway [93]. Currently, the first-generation mTOR inhibitors, including everolimus and sirolimus, are approved for treating BC. However, PI3K inhibitors targeting TNBC are still in phase I clinical trials [70].

Dual PI3K/mTOR inhibitor therapy is reported to be more efficient than single inhibitors [94]. Dual PI3K/mTOR inhibitors, such as apitolisib, suppressed human glioblastoma cell growth and induced apoptosis [95]. B7-H3 can promote resistance to traditional cancer therapy in a variety of tumors, and knocking out B7-H3 has been shown to increase the sensitivity of TNBC cells to everolimus [96, 97]. Dual PI3K/mTOR inhibitors are considered critical in cancer therapy, and many dual PI3K/mTOR inhibitors are available and in use, such as dactolysisib, sarmotolysisib, and voltaricoxib [94].

PI3K/AKT/mTOR oncogenic signaling pathways often induce cancer progression and are associated with resistance to targeted anticancer therapies, and more research is still needed on the effectiveness of related inhibitors [98].

### Epidermal growth factor receptor (EGFR) signaling pathway inhibitors

EGFR is a tyrosine kinase receptor. It's reported that EGFR was an efficient therapeutic target in 89% of TNBC patients, especially for BL2 subtype tumors with overexpression of EGFR [99]. Moreover, EGFR predicts recurrence-free survival and OS in BC patients [100].

EGFR targeting has been approved for treating cancer patients, including tyrosine kinase inhibitors (TKIs) gefitinib and monoclonal antibodies [101] (Fig. 2C, Table 4). Gefitinib inhibits BC cell proliferation and increases the cytotoxicity of carboplatin and docetaxel [102]. In addition, combining three inhibitors, gefitinib, carboplatin, and docetaxel, may synergistically increase cytotoxicity in TNBC cells [103]. However, the reported failure of combination therapy with EGFR TKIs and monoclonal antibodies led to combination therapy with monoclonal antibodies and chemotherapeutic agents, which was a more effective therapeutic strategy. For example, in a clinical trial, cetuximab combination with carboplatin or cetuximab with cisplatin doubled pCR and prolonged PFS and OS in metastatic TNBC patients [104, 105]. Moreover, HOMER3 promoted β-catenin activation through growth factor stimulation, which in turn facilitated the progression of TNBC cells [106]. Sustained activating EGFR/KRAS/SIAH pathway has contributed to chemoresistance in TNBC, and further exploration of chemoresistance will provide new insight for future treatment of TNBC [107].

It has been shown that targeting gefitinib and everolimus can inhibit the activation of the PI3K/AKT/mTOR signaling pathway, thereby blocking cancer cell cycle progression and promoting apoptosis in TNBC cells [108].

### Fibroblast growth factor receptor (FGFR)

Fibroblast growth factor receptors are activated by binding to various fibroblast growth factors and regulate numerous cellular processes. Over-activation of FGFR signaling is observed in some cancers and FGFR has interaction with hormone receptor signaling [109, 110]. There is the amplification of FGFR1 or FGFR2 in TNBC, and FGFR1 activation has been linked to OS prognosis [111–113]. Turner et al. found that FGFR1 or FGFR2-amplified TNBC cell lines were highly sensitive to the FGFR inhibitor PD173074 [114]. Dovitinib, an FGFR1/2 inhibitor, restrained the proliferation of FGFR-amplified BC cell lines [115]. A clinical conversion trial displayed gastric cancer patients with high FGFR2-amplified had higher pCR than the selective FGFR inhibitor AZD4547 [116]. But a study demonstrated that only 1 in 8 breast cancer patients with FGFR1 amplification responded to AZD4547 treatment. These data suggest that FGFR targeting has shown promising results in breast cancer, especially when FGFR is amplified [117] (Fig. 3).

Currently, some clinical trials are ongoing and enrolled patients must undergo molecular pre-screening to ensure the inclusion of patients associated with FGFR pathway activation.

### Vascular endothelial growth factor receptor (VEGFR)

The continuous formation of tumor blood vessels provides sufficient nutrients for tumorigenesis and progression of TNBC [118–120]. Therefore, anti-VEGF treatment can inhibit tumor growth (Fig. 2D, Table 5).

**Table 5:** The clinical trials of VEGF/VEGFR inhibitors in TNBC

Drug name	Com	Num	Regiments	Ph	State	Object	POM	NCT number
Bevacizumab		54	15 mg/ kg	II	Completed	mTNBC	PFS	NCT03577743
Bevacizumab		2591	5 mg/kg q1w	III	Completed	TNBC	IDFS	NCT00528567
Bevacizumab	Albumin-bound paclitaxel	128	Bevacizumab: 7.5 mg/kg iv q3w Albumin-bound paclitaxel: 260 mg/m^2^ iv q3w	II	Recruiting	mTNBC	PFS	NCT05192798
Bevacizumab	Tirelizumab	15	Bevacizumab: 7.5 mg/kg iv q3w Tirelizumab: 200 mg iv q3w	II	Recruiting	mTNBC	ORR	NCT05303038
Bevacizumab	Liposomal doxorubicin hydrochloride/ Everolimus	17	Bevacizumab: iv over 90 min on day 1 Liposomal doxorubicin hydrochloride: iv over about 3 h on day 1 Everolimus: po qd on days 1–21	II	Active, not recruiting	Advanced TNBC	pCR	NCT02456857
Bevacizumab	Paclitaxel/ Docetaxel	49	Bevacizumab: 10 mg/kg iv q2w Paclitaxel q1w or docetaxel q3w	IV	Completed	TNBC	PFS	NCT01094184
Bevacizumab	Docetaxel, Carboplatin	45	Bevacizumab: 7.5 mg/kg iv q3w Docetaxel iv and carboplatin iv	II	Completed	TNBC	pCR	NCT01208480
Bevacizumab	Nab-paclitaxel, erlotinib	59	Bevacizumab: iv over 30–90 min on days 1,15 Nab-paclitaxel: iv on days 1, 8, and 15 erlotinib hydrochloride: po qd	II	Completed	TNBC	PFS	NCT00733408
Bevacizumab	Abraxane, Carboplatin	41	Bevacizumab: 10 mg/kg iv on days 1,15 Abraxane: 100 mg/m^2^ iv over 30 min on days 1,8,15 Carboplatin: AUC = 2 iv over 15 min on days 1,8,15	II	Completed	mTNBC	CBR	NCT00479674
Apatinib	Camrelizumab	58	Apatinib:250 mg po qd Camrelizumab: 200 mg iv on day 1	II	Recruiting	TNBC	pCR	NCT05556200
Apatinib	Camrelizumab and nab-paclitaxel	35	Apatinib:250 mg po qd Camrelizumab: 200 mg iv q2w Nab-paclitaxel: 125 mg/m^2^ iv q1w	II	Not yet recruiting	TNBC	pCR	NCT05447702
Apatinib	Paclitaxel and Carboplatin	29	Apatinib: 250 mg po qd on day 1-14 Paclitaxel: 175 mg/m^2^ on day 1 Carboplatin: AUC = 4 on day 1,14	II	Unknown	TNBC	pCR	NCT03735082
Apatinib	Capecitabine	80	Apatinib: 425 mg on day 1-21 Capecitabine: 1000 mg/m^2^ bid on day 1-14	II	Recruiting	Advanced TNBC	PFS	NCT03775928
Apatinib	Paclitaxel	20	Apatinib: 500 mg po qd 12 weeks Paclitaxel: 80 mg/m^2^ on day 1 q1w	II	Recruiting	Advanced TNBC	ORR	NCT03348098
Apatinib	Albumin paclitaxel and carboplatin	60	Apatinib: 250 mg po on day 1–21 Albumin paclitaxel: 260 mg/m^2^ iv on day 1 Carboplatin: AUC = 5–6 iv on day 1	II	Recruiting	TNBC	pCR	NCT03650738
Apatinib	Camrelizumab and Eribulin	46	Apatinib: 250 mg/d po on day 1-21 Camrelizumab: 200 mg iv q3w Eribulin: 1.4 mg/m^2^ iv on day 1, 8 q3w	II	Active, not recruiting	Advanced TNBC	ORR	NCT04303741

mTNBC: metastatic triple negative breast cancer; PFS: progression-free survival; IDFS: invasive disease-free survival; ORR: overall response rate; pCR: pathologic complete response; CBR: clinical benefit rate

Currently, the commonly used anti-VEGF drug is bevacizumab. A Phase III trial, RIBBON 1, demonstrated that combining bevacizumab with conventional capecitabine, anthracycline, or taxane improved PFS in mTNBC patients [121]. Subsequently, the trial further analyzed the effectiveness of bevacizumab in mTNBC patients. Results have prolonged mPFS (6.0 m vs 2.7 m) in TNBC patients with bevacizumab arm versus placebo arm, and a trend toward improvement in patient OS [122].

The GeparSixto and GeparQunito trials combining bevacizumab with neoadjuvant chemotherapy for treating TNBC patients showed significant improvement in pCR in TNBC patients [123–125]. However, the results from the BEATRICE trial demonstrated bevacizumab failed to improve OS in early TNBC patients [126]. The FDA withdrew bevacizumab for treating BC because of inconsistencies in treating TNBC patients.

Apatinib has shown antitumor effects by inhibiting VEGFR signaling in TNBC cells [127]. The LANCET trial, administrating apatinib and neoadjuvant chemotherapy (apatinib and docetaxel in combination with epirubicin and cyclophosphamide) in TNBC patients showed excellent efficacy and controlled toxicity [128]. Furthermore, the NAN trial suggested that adding apatinib to advanced TNBC patients who had failed first/second-line therapy improved their PFS with good safety [129]. Liu et al. verified that the combination of camrelizumab and apatinib could effectively improve ORR in patients with advanced TNBC [130]. The above trials suggested that apatinib was effective in the treatment of some patients with TNBC.

### Notch signaling pathway inhibitors

Morgan et al. described the family of transmembrane ligands and receptors named Notch. The pathway includes four Notch receptors, namely Notch-1, 2, 3, and 4 receptors, and five ligands, namely Jagged-1, Jagged-2, Delta-1, Delta-3, and Delta-4 [131, 132]. It has been reported that Delta-1 and Jagged-1 are overexpressed in BC, while Notch-1 is also important for tumorigenesis of BC in the form of oncogenic Ras downstream effectors [133, 134]. Many transcription factors encode genes that are associated with tumorigeneses in Notch signaling, including the HES family and HEY family [131]. Notch signaling pathway was essential in the progression of many types of cancer, such as hematological malignancies, BC, lung cancer, hepatocellular carcinoma, pancreatic cancer, and colorectal cancers [131, 135]. Several studies have shown that Notch-3 and Notch-4 have been associated with tumor initiation and proliferation [136]. However, overexpression of Notch-2 appears to be a protective factor in TNBC cell lines [137]. Moreover, the Notch pathway plays a relevant role in BC stem cell maintenance and expansion, and Notch receptor expression and activation are closely associated with the aggressiveness, clinicopathology, and biological phenotype (e.g., invasiveness and chemotherapy resistance) of TNBC [138].

Since the Notch receptor is overexpressed in TNBC, researchers suggest that monoclonal antibodies (mAb) target the receptor as a prospective way to treat TNBC [139]. Current studies on mAb inhibition of Notch-1 signaling indicated that it could effectively reduce the expression of HES and HEY-L families in TNBC cells, inhibit cell proliferation, and promote treatment induced-apoptosis [140]. In addition, treatment with DLL4 (delta-like ligand 4) monoclonal antibody was effective in TNBC [141]. Drugs that interfere with the Notch signaling pathway act by blocking the level of hydrolytic cleavage of the multimeric γ-secretase complex in the cytoplasm and these agents are therefore referred to as γ-secretase inhibitors [142]. Unfortunately, many agents that block the Notch pathway are not approved by FDA.

In summary, abnormal activating of the Notch signaling pathway is associated with malignant biological behavior and prognosis in TNBC. Therefore, an in-depth exploration of the role played by TNBC in this signaling pathway will further improve the understanding of TNBC pathogenesis and thereby explore new targeted therapeutic strategies (Table 4).

### STAT3 signaling pathway inhibitors

STAT3 plays an oncogenic effect by participating in the regulation of the expression of genes connected to the malignant biological behavior of tumors [143]. Its constitutive activation is mainly due to the dysregulation of upstream signaling, usually mediated by several cytokines and growth factors, such as IL-6 and EGF [144, 145]. STAT3 is important in BC stem cell progression, maintaining gene expression associated with stem cell phenotype [146] (Fig. 3).

The activation of STAT3 or inhibition of ROS promotes radio-resistance in TNBC, while clonidine plays an effective sensitizer by inhibiting STAT3 and increasing ROS expression in vitro from TNBC. These results showed clonidine combined with irradiation can be an effective approach to ameliorate radiation-resistance in TNBC cells to improve therapeutic efficacy [147]. WZ-2–033, a novel STAT3 inhibitor, inhibits pY705-STAT3 phosphorylation, thereby reducing STAT3-dependent transcriptional activity and suppressing STAT3 expression from downstream genes. WZ-2–033 significantly suppressed the proliferation and tumorigenicity of TNBC in vivo and in vitro via blocking STAT3 activation [148].

### Transforming growth factor (TGF) -β inhibitors

TGF-β1 is a member of the TGF-β superfamily [149]. It has been clarified that TGF-β is negatively associated with the prognosis of TNBC patients [150]. Xu et al. proposed that TGF-β was crucial in TNBC drug resistance, regulating tumor cell stemness, epithelial-mesenchymal transition, and apoptosis [149]. TGF-β inhibited the initiation and proliferation of chemotherapy-resistant tumor-initiating cells. This lays the groundwork for the adoption of combination chemotherapy in TNBC patients [151]. TGF-β overexpressed in TNBC cells, which leads to tumor metastasis. It’s suggested that TGF-β inhibitors were essential for patients with metastases [152]. Besides, TGF-β also causes immune evasion and immunotherapy resistance of TNBC [153–155]. In the tumor microenvironment, regulatory T cells, macrophages, MDSC, and fibroblasts co-express TGF-β1 and PD-L1. Bi-functional fusion protein Bintrafusp alfa was designed for simultaneous inhibition of two immunosuppressive pathways in the tumor microenvironment. The study by Lan demonstrated that Bintrafusp alfa more effectively blocked TGF-β and showed superior antitumor response compared to single-agent therapy [156]. Moreover, Yi et al. constructed an anti-TGF-β/PD-L1 bispecific antibody YM101, which promoted T-cell infiltration and exhibited stronger inhibitory tumor activity in TNBC [157–160]. In view of the role of TGF-β in TNBC, TGF-β inhibitors may be an effective treatment for TNBC (Fig. 3, Table 4).

### Epigenetic modifications

Epigenetic modifications, such as DNA methylation and histone modification, are involved in the development of various cancers, and it has also been hypothesized that this may be a therapeutic strategy for TNBC [161, 162].

The ER is present in TNBC but is silenced due to the demethylation of ER CpG islands and reduced histone activity. Reactivation of the ER may be a therapeutic strategy for TNBC. Histone deacetylase (HDAC) inhibitors and demethylation inhibitors have been reported to reactivate ER [163]. Tan et al. found that the RNA N6 -methyladenosine reader YTHDC1 promotes metastasis in TNBC cells; therefore, targeting the YTHDC1/m^6^A/SMAD3 axis could be a potential therapeutic strategy for TNBC [164]. Decitabine induces DNA hypomethylation and has been approved by the FDA for treating myelodysplastic syndromes. It also plays a role in the treatment of patients with BC. The related clinical trial is ongoing, with results to be announced. In addition, Jiang et al. found that compound A6, which targets both HDAC and G-quadruplex (G4), significantly inhibited the proliferation of TNBC cells and demonstrated a favorable safety profile in a mouse model [165]. Moreover, the combination of HDAC inhibitors and ionizing radiation may benefit patients with TNBC [166]. The combination of HDAC inhibitors and letrozole showed favorable efficacy in patients with mBC [167]. There are several other HDAC inhibitors currently in clinical trials, such as belinostat, chidamide, romidepsin, and entinostat [168, 169].

Overall, using HDAC inhibitors or DNA methylation inhibitors may be a promising therapeutic strategy for patients with TNBC.

## Immunotherapy

The 2023 ASCO conference unveiled the results of the TORCHLIGHT clinical trial, which demonstrated that the combination of toripalimab and nab-paclitaxel can significantly extend the PFS of patients with stage IV breast cancer or recurrent and metastatic TNBC [170, 171]. Furthermore, the findings from the 'FUTURESUPER' clinical trial indicated that immunotherapy based on molecular subtypes, like IM of TNBC, can improve the outcome of patients [172].

Strategies for TNBC immunotherapy include increasing the antigen-presenting capacity of dendritic cells and activating effector T lymphocyte function, suppression of regulatory T lymphocytes and myeloid-derived suppressor cells, upregulating relevant cytokines to reverse the tumor suppressive microenvironment, and promoting antitumor immune responses to kill tumor cells [18, 173–175]. For example, ICIs, CAR-T, and tumor vaccines (Fig. 2F).

The programmed death receptor (PD-1) and its ligand PD-L1 are the current topics in targeted therapies, which lead to sustained clinical relief in various types of cancer, including non-small cell lung cancer, hepatocellular carcinoma, renal cell carcinoma, and others [176–182]. Compared with other types of BC, TNBC shows a higher tumor mutation burden, higher levels of PD-L1 expression, and more immune cell infiltration into the tumor microenvironment. Hence, TNBC is the most immunogenic subtype capable of benefiting from immunotherapy. The IM type represents about 24% of TNBC and is more sensitive to immunotherapy due to its characteristic activation of immune regulatory pathways [183]. At present, there are many ongoing clinical trials for TNBC patients (Table 6).

**Table 6:** The clinical trials of PD-1/PD-L1 inhibitors for TNBC

Drug name	Com	Name	Num	Regiments	Phase	Status	Object	POM	NCT number
Pembrolizumab (MK-3475, KEYTRUDA®)		KEYNOTE-086	254	200 mg iv on day 1 of q3w for up to 35 cycles	II	Completed	mTNBC	ORR, AEs	NCT02447003
Pembrolizumab		KEYNOTE-012	297	10 mg/kg iv q3w	I	Completed	mTNBC	ORR, AEs	NCT01848834
Pembrolizumab		TAPUR	28	2 mg/kg or 200 mg iv of q3w	II	Recruiting	mTNBC	ORR	NCT02693535
Pembrolizumab		SWOG 1418	1155	Iv over 30 min on days 1 and 22. Cycles repeat every 42 days for 52 weeks	III	Active, not recruiting	TNBC	iDFS	NCT02954874
Pembrolizumab	Chemotherapy (Capecitabine/Eribulin/Gemcitabine/Vinorelbine)	KEYNOTE-119	622	Pembrolizumab: 200 mg iv q3w for up to 35 administrations Chemotherapy: as TPC in accordance with local regulations and guidelines	III	Completed	Advanced or mTNBC	OS	NCT02555657
Pembrolizumab	Chemotherapy (Nab-paclitaxel /Doxorubicin/ Cyclophosphamide)	KEYNOTE-173	60	Pembrolizumab: 200 mg iv q3w Nab-paclitaxel: 125 or 100 mg/m^2^ iv q3w doxorubicin: 60 mg/m^2^ iv q3w Cyclophosphamide: 600 mg/m^2^ q3w	I	Completed	TNBC	DLTs, AEs	NCT02622074
Pembrolizumab	Gemcitabine/carboplatin	KEYNOTE 355	882	Pembrolizumab: 200 mg iv on day 1 of each 21-day cycle Gemcitabine/carboplatin: 1000 mg/m^2^ (gemcitabine) and an AUC 2 (carboplatin) on days 1 and 8 of each 21-day cycle	III	Active, not recruiting	mTNBC	AEs, PFS, OS	NCT02819518
Pembrolizumab	Paclitaxel plus carboplatin	KEYNOTE-522	1174	Pembrolizumab: 200 mg iv q3w Paclitaxel + carboplatin: q3w × 4 cycle Each cycle is 21 days	III	Active, not recruiting	TNBC	pCR, EFS	NCT03036488
Pembrolizumab	Carboplatin and gemcitabine/ olaparib	KEYLYNK-009	460	Pembrolizumab: 200 mg iv on day 1 of each 21-day cycle Carboplatin: AUC 2 with gemcitabine 1000 mg/m^2^ iv on days 1 and 8 of each 21-day cycle Olaparib: 300 mg qd po	II	Active, not recruiting	mTNBC	PFS, OS	NCT04191135
Pembrolizumab	Eribulin	ENHANCE 1	258	Pembrolizumab: 200 mg iv on day 1 of each 21-day cycle Eribulin: 1.4 mg/m^2^ iv on day 1 and 8 of each 21-day cycle	I/II	Completed	Advanced or mTNBC	ORR	NCT02513472
Pembrolizumab	Ladiratuzumab vedotin	SGNLVA-002	211	Pembrolizumab: iv q3w ladiratuzumab vedotin: iv	I/II	Recruiting	Advanced or mTNBC	DLTs, AEs, ORR	NCT03310957
Pembrolizumab	Dinaciclib		32	Pembrolizumab: 200 mg iv on day 1 q3w Dinaciclib: 12 mg/m^2^ day 1 and 8 of a 21 days cycle by 2-h iv	I	Completed	Advanced or metastatic TNBC	MTD, DLTs	NCT01676753
Pembrolizumab	Enobosarm		18	Pembrolizumab: 200 mg iv over 30 min on day 1 Enobosarm: po qd on days 1–21	II	Active, not recruiting	mTNBC	pCR	NCT02971761
pembrolizumab	Imprime	IMPRIME 1	64	Pembrolizumab: 200 mg iv over 30 min on Day 1 of q3w Imprime: 4 mg/kg iv over a 2-h infusion time on days 1, 8 and 15 of q3w treatment cycle	I	Completed	Advanced or mTNBC	ORR	NCT02981303
pembrolizumab	Paclitaxel/ Doxorubicin/ Cyclophosphamide	ISPY-2		Pembrolizumab: 200 mg iv cycles 1,4,7,10 Paclitaxel: 80 mg/m^2^ iv cycles 1–12 Doxorubicin: 60 mg/m^2^ iv every 2 or 3 weeks for 4 cycles Cyclophosphamide: 600 mg/m^2^ iv every 2 or 3 weeks for 4 cycles	III	Recruiting	Stage II–III TNBC	pCR	NCT01042379
Atezolizumab			661	0.01 mg/kg iv q3w	I	Completed	Advanced or mTNBC	DLTs, MTD	NCT01375842
Atezolizumab		JAVELIN	168	1.0 mg/kg once q2w	I	Completed	Advanced or mTNBC	DLTs	NCT01772004
Atezolizumab	Nab-paclitaxel/ Doxorubicin/ Cyclophosphamide	IMpassion031	333	Atezolizumab: 840 mg iv q2w Nab-paclitaxel: 125 mg/m^2^ iv every week for 12 weeks Doxorubicin: 60 mg/m^2^ iv Cyclophosphamide: 600 mg/m^2^ iv q2w	III	Completed	TNBC	pCR	NCT03197935
Atezolizumab	Nab-paclitaxel	IMpassion130	902	Atezolizumab: 840 mg iv on days 1 and 15 of each 28-day cycle Nab-Paclitaxel: 100 mg/m^2^ iv on days 1, 8, and 15 of each 28-day cycle	III	Completed	Advanced or mTNBC	PFS, OS	NCT02425891
Atezolizumab	Paclitaxel	IMpassion131	653	Atezolizumab: 840 mg iv on days 1 and 15 (± 3 days) of every 28-day cycle Paclitaxel: 90 mg/m^2^ iv on days 1, 8, and 15 of every 28-day cycle	III	Completed	Advanced or mTNBC	PFS	NCT03125902
Atezolizumab	Capecitabine or gemcitabine/carboplatin	IMpassion132	572	Atezolizumab: 1200 mg iv Gemcitabine: 1000 mg/m^2^ on days 1 and 8 of q3w Capecitabine: 1000 mg/m^2^ po bid on days 1 to 14 q3w	III	Recruiting	Advanced or mTNBC	OS	NCT03371017
Atezolizum	Nab-paclitaxel plus cobimetinib +	COLET	169	Atezolizumab: 840 mg iv q2w on days 1 and 15 Paclitaxel: 80 mg/m^2^ iv on day 1, 8, 15 Cobimetinib: 60 mg/d on day 3–23 Each 28-day treatment cycle	II	Completed	Advanced or mTNBC	PFS, ORR	NCT02322814
	Ipatasertib/ Paclitaxel		140	Atezolizumab: 840 mg iv q2w on days 1 and 15 Ipatasertib: 400 mg/d po on days 1–21 Paclitaxel: 80 mg/m^2^ iv on day 1, 8, 15	I	Completed	Advanced or mTNBC	pCR	NCT03800836
Atezolizumab	Paclitaxel /Doxorubicin	IMpassion 030	2300	Atezolizumab: 840 mg iv q2w Paclitaxel: 80 mg/m^2^ qw for 12 weeks Doxorubicin: 60 mg/m^2^ iv q2w	III	Active, not recruiting	Stage II-III TNBC	iDFS	NCT03498716
Atezolizumab	Capecitabine	MIRINAE	284	Atezolizumab: 1200 mg iv q3w Capecitabine: 2500 mg/m^2^/d day 1–14, q3w for 8 cycles	II	Recruiting	TNBC	iDFS	NCT03756298
Avelumab		A-BRAVE	474	10 mg/kg iv q2w for 1 year	III	Active, not recruiting	TNBC	DFS	NCT02926196
Durvalumab (MEDI4736)	Nab-paclitaxel	GeparNuevo	174	MEDI4736: 1.5 g iv q4w Nab-Paclitaxel 125 mg/m^2^ qw for 12 weeks	II	Completed	TNBC	pCR	NCT02685059
Durvalumab	Olaparib	MEDIOLA	264	Olaparib: bid starting on week 1 day 1 MEDI4736: q4w starting on week 5 day 1	I/II	Active, not recruiting	gBRCA-mBC	safety and tolerability; ORR	NCT02734004
Nivolumab		TONIC	84	Nivolumab: 3 mg/kg q2w	II	Active, not recruiting	Advanced or mTNBC	PFS	NCT02499367
Nivolumab	Pembrolizumab	TOPACIO/KEYNOTE-162	122	Niraparib: 300 mg/d PO on days 1–21 Pembrolizumab: 200 mg iv on day 1 of each 21-day cycle	I/II	Completed	Advanced or mTNBC	DLTs, ORR	NCT02657889
Camrelizumab (SHR-1210)	Apatinib		40	SHR-1210: 3 mg/kg iv q2w Apatinib: 250 mg/d po day 1–14	II	Completed	mTNBC	ORR	NCT03394287

mTNBC: metastatic triple negative breast cancer; TNBC: triple negative breast cancer; HER2-: HER2 negative; mBC: metastatic breast cancer; gBRCA1/2 m: germline BRCA1/2 mutated; ORR: overall response rate; AEs: adverse events; iDFS: invasive disease-free survival; OS: overall survival; DLTs: dose-limiting toxicity; PFS: progression-free survival; EFS: event-free survival; MTD: maximum tolerated dose; pCR: pathologic complete response; DFS: disease-free survival

PD-L1 links to PD-1 on the surface of tumor-infiltrating lymphocytes and inhibits lymphocyte function and cytokine release, causing the immune escape from cancer cells [184–186]. Ali et al. detected PD-L1 expression in BC at about 6.3% in 3,916 tumor samples, increasing to 19% in TNBC [187]. Mittendorf et al. also obtained the same results as Ali et al. by using the cancer genome atlas (TCGA) RNA sequencing [188]. The above results suggested that inhibition of PD-1 binding to PD-L1 might be a promising approach for TNBC.

### ICIs

Currently, ICIs include PD-1 and PD-L1 inhibitors, which are widely utilized in the clinic, such as pembrolizumab, atezolizumab, durvalumab, and nivolumab [189]. Moreover, drugs related to new immunotherapeutic targets, for example, LAG3, TIM3, and ICOS are under development [190, 191].

Pembrolizumab, a PD-1 inhibitor, has demonstrated antitumor activity and manageable safety in KEYNOTE-012 and KEYNOTE-086 for pembrolizumab monotherapy in mTNBC [192–194]. The KEYNOTE-119 trial displayed that administration of pembrolizumab monotherapy didn’t prolong the comparison of OS with chemotherapy in patients with mTNBC, but in the pembrolizumab group, drug efficacy increased with increasing PD-L1 expression, demonstrating that high PD-L1 expression may be related to the CBR of pembrolizumab [195]. KEYNOTE-355 was launched to assess the effectiveness of pembrolizumab plus neoadjuvant chemotherapy as a first-line therapy for patients with early-stage TNBC. It demonstrated that pembrolizumab combined with chemotherapy resulted in a higher percentage of pCR in patients in the PD-L1 overexpression group compared to neoadjuvant chemotherapy [196, 197]. The above studies suggested that it is worth exploring the value of combining conventional treatment with immunotherapy for patients with TNBC.

The subsequent KEYNOTE-173 trial, Phase II trial I-SPY2, and Phase III trial KEYNOTE-522 combination with chemotherapy resulted in better antitumor activity, significantly improved pCR rates, and extended event-free survival in TNBC patients [198–200]. These trials confirmed the value of pembrolizumab in treating TNBC with neoadjuvant therapy [196, 201]. The AGO-B-041 trial demonstrated combined pembrolizumab with nab-paclitaxel in TNBC patients with a pCR of 59.3% [202].

The FDA has approved pembrolizumab for postoperative adjunctive therapy in TNBC patients or further chemotherapy in patients with locally recurrent, unresectable, or mTNBC with high PD-L1 expression [203].

The IMpassion031 trial displayed that combining atezolizumab with a standard chemotherapy regimen meaningfully increased pCR in TNBC patients with a good safety profile [204]. The FDA has approved neoadjuvant therapy of atezolizumab monotherapy or plus nab-paclitaxel to treat patients with metastatic or locally advanced TNBC expressing PD-L1 [205]. Another trial assessed the effectiveness of atezolizumab added to nabilone paclitaxel in TNBC patients. It clarified notably longer PFS in patients treated with the combination and a more pronounced OS benefit in the high PD-L1 expression group [206–208]. In a similar trial, IMpassion131, atezolizumab in combination with paclitaxel didn’t improve PFS and OS of TNBC patients [209]. The impact of the difference between the two assays deserves further exploration.

​ Durvalumab is a PD-L1 monoclonal antibody. The GeparNUEVO trial used durvalumab in the neoadjuvant setting for TNBC and observed an increase in pCR, improvement in iDFS and DDFS, and a favorable trend in OS [210, 211]. Another trial, SAFIRO2-BREAST IMMUNO, which examined the efficacy of durvalumab in metastatic BC patients, showed that durvalumab didn’t prolong PFS and OS in BC patients, but significantly prolonged OS in TNBC patients [211].

### Other methods of immunotherapy

Chimeric antigen receptor (CAR) T cells therapy utilizes genetic engineering to modify a patient's peripheral T-cells, giving them the characteristics to target and identify tumor cells. After in vitro expansion and culture, cells were transfused into patients to precisely kill tumors [212–214]. CAR-T therapy is known to be effective in hematologic tumors, but its efficacy in solid tumors is still being explored [215–217]. Currently, CAR-T therapy targeting ROR1 and MUC1 are promising therapeutic targets in TNBC [218]. Harrasser et al. designed CAR-T targeting ROR1, which demonstrated favorable antitumor activity in vivo models of TNBC with a good safety profile [219]. The related clinical trials are ongoing. ​CAR-natural killer (NK) cells targeting EGFRvIII are available for treating BC, and preclinical studies with tissue factor-targeted CAR-NK cells as monotherapy in TNBC have shown promising efficacy [220, 221]. Moreover, EGFR-targeted CAR-T showed potential antitumor effects in TNBC*.* It may be a prospective immunotherapy strategy for TNBC [222]. The promising effect of CAR-T therapy in TNBC deserves further studies (Table 7).

**Table 7:** The clinical trials of tumor vaccine and CAR-T in TNBC

Drug name	Com	Num	Regiments	Phase	Status	Object	POM	NCT number
Neoantigen vaccine	Nab-paclitaxel plus Durvalumab	70	Vaccine and poly-ICLC SC: on days 1, 4, 8, 15, 22, 50, and 78 Nab-paclitaxel: iv on days 1, 8, and 15 of each cycle Durvalumab: iv on day 1 of each cycle	II	Recruiting	Advanced or mTNBC	PFS	NCT03606967
anti-meso CAR-T cells		20	a standard 3 + 3 dose escalation approach	I	Unknown	TNBC	AEs	NCT02580747
cMet RNA CAR T cells		6	3 × 10^7 or 3 × 10^8 cells	I	Completed	mTNBC	AEs	NCT01837602
CART-TnMUC1 cells		112	Single iv administration	I	Active, not recruiting	HER2- TNBC	DLTs	NCT04025216
EGFR/B7H3 CAR-T cells		30	2 × 10^6/kg CAR-T cells	I	Recruiting	TNBC	AEs	NCT05341492
NKG2DL-targeting CAR-grafted γδ-T Cells		10	"3 + 3" dose escalation study design ranging from 3 × 10^8—3 × 10^9 CAR-γδ-T cells. Each cycle of therapy will consist of 4 iv, given 7 days apart	I	Unknown	TNBC	DLTs	NCT04107142
ROR1-targeted CAR T cells (LYL797)		54	NA	I	Recruiting	TNBC	DLTs, TEAEs	NCT05274451
ROR1-CAR-T cell		21	ROR1 CAR-specific autologous T-lymphocytes IV over 20–30 min	I	Terminated	TNBC	AEs	NCT02706392
PD-1^+^ TILs		20	5 to 10 mg/mL/min	I/II	Not yet recruiting	PD1^+ ^TNBC	AEs, PFS	NCT05451784
TC-510		115	NA	I/II	Recruiting	TNBC	DLTs, ORR	NCT05451849
BT-001	Pembrolizumab	48	BT-001: administered at different dose Pembrolizumab: 200 mg iv q3w	I/II	Recruiting	TNBC	AEs	NCT04725331

CAR-T: chimeric antigen receptor-T cell; mTNBC: metastatic triple negative breast cancer; HER2-: HER2 negative; mBC: metastatic breast cancer; gBRCA1/2 m: germline BRCA1/2 mutated; ORR: overall response rate; AEs: adverse events; PFS: progression-free survival; DLTs: dose-limiting toxicity; TEAEs: treatment emergent adverse events; ROR1: receptor tyrosine kinase like orphan receptor 1; PD-1: programmed cell death protein 1; EGFR: epidermal growth factor receptor; NKG2DL: natural killer group 2, member D

A tumor vaccine is an emerging immunotherapy strategy that works by introducing a tumor antigen into a patient’s body, activating or enhancing the body's immune system, and producing a valid antitumor immune response that kills or eliminates tumor cells [223]. At present, TNBC vaccines in development mainly include dendritic cell vaccines, peptide vaccines, and modified exosome vaccines [224–228]. GM-CSF is a tumor vaccine adjuvant in ongoing clinical trials for immunotherapy of BC [229]. BT-001 is an ongoing TNBC-related clinical trial as a lysovirus vaccine expressing cytotoxic T lymphocyte-associated antigen-4 antibodies and GM-CSF [230, 231] (Table 7).

​In addition to this, considerable data have demonstrated that targeting nucleotide metabolism could enhance the antitumor immune response [232–234]. The efficacy of targeting nucleotide metabolism in combination with immunotherapy versus immunotherapy monotherapy for TNBC will be compared in ongoing clinical trials [235].

### Combination therapy

In addition to the above treatment strategies of combining immunotherapy with chemotherapy, there are some combination approaches to maximize the benefits of cancer immunotherapy to enhance the efficacy of ICIs [236–238] (Table 7).

Combining ICIs with DNA damage repair inhibitors, including PARP inhibitors, is a promising strategy for BC patients with BRCA mutations [239]. In the TNBC tumor model, niraparib activated interferon signaling and enhanced the anti-tumor activity of the anti-PD-1 antibody BioXCell RMP1-14 in TNBC. A synergistic suppressed tumor effect was revealed when nirapanib was administered with BioXCell RMP1-14 [240]. Intriguingly, KEYNOTE-162 evaluated the efficacy of combining niraparib with pembrolizumab in advanced or mTNBC patients. Combining niraparib with pembrolizumab has displayed favorable efficacy and safety in TNBC patients with BRCA mutations [241]. Another phase II trial, I-SPY2, indicated that adding durvalumab in combination with olaparib to the standard chemotherapy raised pCR to 20% in TNBC patients [242]. ​TNBC patients who received nab-paclitaxel plus atezolizumab were able to extend PFS, but in the IMpassion131 clinical trial, it was disappointing that the combination of paclitaxel and atezolizumab did not improve PFS or OS in TNBC patients [209].

According to the favorable results of the above clinical trials or related studies, immunotherapy is expected to bring benefits to TNBC patients.

## Antibody–drug conjugate (ADC)

**Fig. 4:**
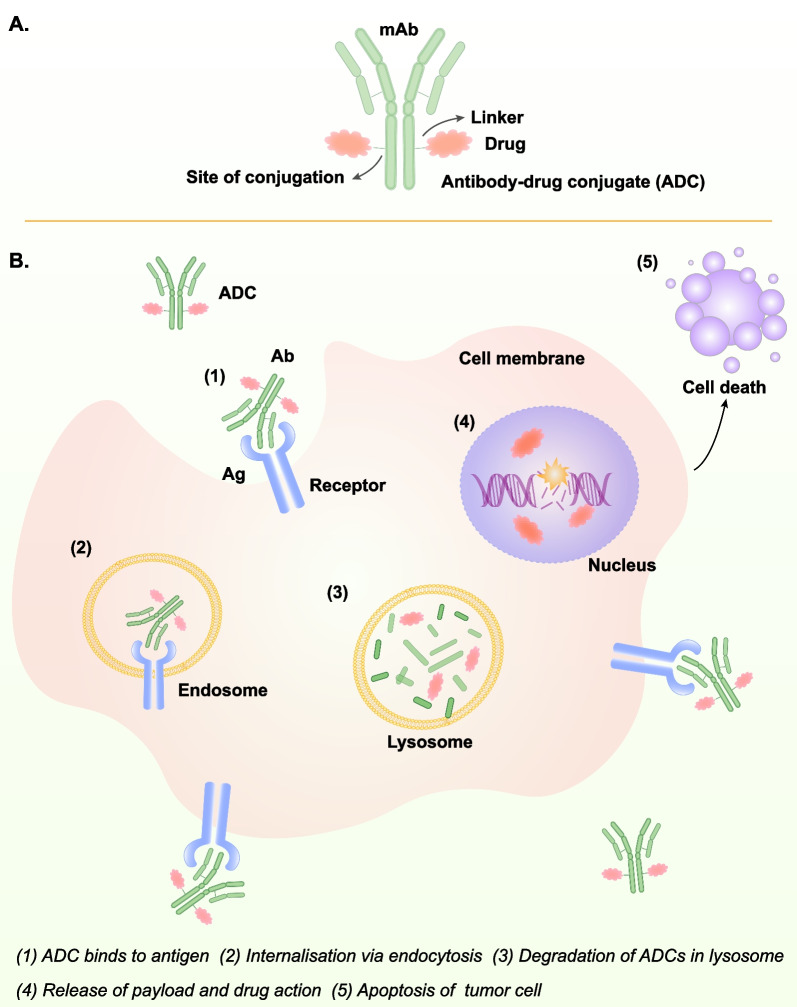
The structure and mechanism of ADC. A. The structure of ADC, B. The mechanism of ADC: (1)-(5). (The action mechanism of ADC was adapted from Fig. 2 in [243])

In recent years, research on ADC is in full swing. ADC mainly uses antibodies as carriers to deliver cytotoxic drugs into tumor cells, breaking double-stranded DNA and further leading to tumor cell death, thus achieving high tolerance and enhanced cytotoxic effects [243] (Figs. 2E, 4). Due to its remarkable clinical efficacy, it provides a new option for tumor patients and further prolongs their survival [244, 245].

Trophoblast cell-surface antigen 2 (Trop-2) is a transmembrane glycoprotein, which is encoded via the TACSTD2 gene, and is highly expressed on TNBC. Overexpression Trop-2 is often predictive of a more aggressive and worse prognosis [246, 247]. Gosartumumab (Sacituzumab govitecan, SG), also known as Trodelvy, consists of SN-38 coupled with humanized Ig G antibody that targets Trop-2. SN-38 is the active metabolite of irinotecan (CPT-11) and functions as a topoisomerase I inhibitor [248]. The IMMU-132–01 trial administrated at least second-line therapy followed by SG therapy in mTNBC patients. The SG group had an ORR of 33.3%, mPFS of 5.5 months, and OS of 13.0 months [249]. The ASCENT trial confirmed that SG significantly improved ORR (35% vs. 5%), PFS (5.6 months vs. 1.7 months), and OS (12.1 months vs. 6.7 months) in mTNBC patients compared to standard chemotherapy regimens and that patients with high TROP-2 expression were more likely to benefit from SG treatment [250]. Moreover, the serial ASCENT trial proved that the PFS and OS of patients with mTNBC who did not respond to chemotherapy significantly improved after SG treatment [251]. The success of the ASCENT study made SG the world's first approved ADC drug for mTNBC by targeting Trop-2 [252, 253].

TROPiCS-02 is an open-label, randomized, multicenter phase III study that evaluates the efficacy and safety of SG versus single-agent chemotherapy in patients with HR + /HER2- who received at least two but no more than four prior chemotherapy regimens for their metastatic disease [254]. Rugo et al. presented the results of the TROPiCS-02 and concluded that SG significantly improved PFS over chemotherapy [255]. The SASCIA trial will determine whether SG can prolong recurrence-free survival in patients with early-stage breast cancer after surgery [256].

Several studies investigating the efficacy of SG for patients with TNBC are underway. These studies assess the efficacy of the agent as neoadjuvant therapy in early TNBC and metastatic cancer in combination with immunotherapy-based regimens or with a PARP inhibitor (Table 8). Collectively, these results suggest that SG is significantly superior to chemotherapy in improving PFS and OS in recurrent and refractory TNBC and heavily pretreated and endocrine-resistant HR^+^/HER2^−^, BC subtypes with limited treatment options and poor prognosis.

**Table 8:** The clinical trials of ADC in TNBC

Drug name	Com	Num	Regiments	Phase	Status	Object	POM	NCT number
PTK7-ADC (PF-06647020)	Gedatolisib	18	PTK7-ADC: 1.4 mg/kg or 2.8 mg/kg on day 1 of every 21 days cycle Gedatolisb: 110 mg or 180 mg iv on day 1, 8, 15 of every 21 days cycle	I	Completed	TNBC	Safety and toxicity	NCT03243331
Sacituzumab Govitecan (SG, Trodelvy, IMMU-132)		80	10 mg/kg iv on day 1 and 8 of a 21-day cycle	II	Active, not recruiting	TNBC	ORR	NCT04454437
SG		52	6 mg/kg iv on day 1 and day 8 of a 21-day cycle	I/II	Active, not recruiting	TNBC	TEAEs, DLTs, ORR	NCT05101096
SG		540	SG: 10 mg/kg on days 1 and 8 of a 21-day cycle	III	Recruiting	TNBC PD-L1 negative	PFS	NCT05382299
SG	Trilaciclib (CDK4/6 inhibitor)	30	SG: 10 mg/kg reconstituted to a concentration of 1.1 mg/mL to 3.4 mg/mL in normal saline Trilaciclib: solution as a 30-min iv to be completed within 4 h prior to the start of SG	II	Active, not recruiting	TNBC	PFS	NCT05113966
SG	Talazoparib (PARP inhibitor)	75	SG: 10 mg/kg iv on days 1 and 8 of a 21 day cycle Talazoparib: qd	I/II	Recruiting	mTNBC	DLTs	NCT04039230
SG	Pembrolizumab	1514	SG: 10 mg/kg iv on days 1 and 8 of 21-day cycles Pembrolizumab: 200 mg iv on day 1 of 21-day cycles for 8 cycles	III	Recruiting	TNBC	iDFS	NCT05633654
SG	Pembrolizumab	110	SG: given on days 1 and 8 of the 21 days cycle Pembrolizumab: given on day 1 of the 21 days cycle	II	Recruiting	TNBC	PFS	NCT04468061
SG	Pembrolizumab	260	SG: 10 mg/kg iv, two days per 21-day cycle Pembrolizumab: 200 mg iv on day 1 of 21-day cycles	II	Recruiting	TNBC	pCR	NCT04230109
SG	Pembrolizumab	440	SG: 10 mg/kg iv on days 1 and 8 of 21-day cycles Pembrolizumab: 200 mg iv on day 1 of 21-day cycles	III	Recruiting	TNBC PD-L1 positive	PFS	NCT05382286
TH1902		70	300 mg/m^2^ iv	I	Active, not recruiting	TNBC	Safety and Tolerability	NCT04706962
Datopotamab Deruxtecan (Dato-DXd, DS-1062a)		118	NA multiple cohorts	I/II	Recruiting	TNBC	ORR	NCT05460273
Dato-DXd		770	All participants enrolled in the dose escalation part	I	Recruiting	TNBC	DLTs, AEs	NCT03401385
Dato-DXd		600	100 mg iv	III	Recruiting	TNBC	PFS	NCT05374512
Dato-DXd	Durvalumab	1075	Dato-DXd: 6 mg/kg iv q3w × 8 cycles Durvalumab: 1120 mg iv q3w × 9 cycles	III	Recruiting	TNBC	iDFS	NCT05629585
T-DXd	Capecitabine	139	T-DXd: 5.4 mg/kg iv q3w Capecitabine: 1000 mg/m^2^ po bid days 1–14 q3w	I	Active, not recruiting	HER2-Low BC	AEs, SAEs	NCT04556773
T-DXd	Durvalumab plus Paclitaxel	139	T-DXd: 5.4 mg/kg iv q3w Durvalumab: 1120 mg iv q3w Paclitaxel: 80 mg/m^2^ iv qw in 3-week cycles	I	Active, not recruiting	HER2-Low BC	AEs, SAEs	NCT04556773
T-DXd	Capivasertib	139	T-DXd: 5.4 mg/kg iv q3w Capivasertib: 400 mg po bid	I	Active, not recruiting	HER2-Low BC	AEs, SAEs	NCT04556773
T-DXd	Fulvestrant	139	T-DXd: 5.4 mg/kg iv q3w Fulvestrant: 500 mg im q4w	I	Active, not recruiting	HER2-Low BC	AEs, SAEs	NCT04556773
T-DXd	Anastrozole	88	T-DXd: 5.4 mg/kg iv q3w Anastrozole: 1 mg/d po	II	Recruiting	HER2-Low BC	pCR	NCT04553770
U3-1402 (Patritumab Deruxtecan)		120	5.6 mg/kg iv on day 1 of q3w	II	Recruiting	mBC	ORR, PFS-6	NCT04699630
CAB-ROR2- ADC (BA3021)	PD-1 inhibitor	420	NA	I/II	Recruiting	TNBC	Safety, ORR	NCT03504488
Vobramitamab duocarmazine (MGC018)		143	3.0 mg/kg iv q3w	I/II	Active, not recruiting	Advanced Solid Tumor	AEs, SAEs, DLTs	NCT03729596

ADC: antibody–drug conjugate; TNBC: triple negative breast cancer; BC: breast cancer; HER2-: HER2 negative; mBC: metastatic breast cancer; gBRCA1/2 m: germline BRCA1/2 mutated; ORR: overall response rate; PFS: progression-free survival; AEs: adverse events; SAEs: serious adverse events; TEAEs: treatment emergent adverse events; DLTs: dose-limiting toxicity; iDFS: invasive disease-free survival; CDK4/6: cyclin-dependent kinases 4/6; pCR: pathologic complete response

TH1902 is a peptide-docetaxel conjugate with a payload of two docetaxel molecules ester-linked to a peptide (TH19P01) designed to recognize sortilin (SORT1). TH1902 is internalized in cancer cells through SORT1 [257]. TH1902 exerts a superior anticancer activity than unconjugated docetaxel in human SORT1-positive ovarian and triple-negative breast cancer xenograft models [257–259]. TH1902 is currently being evaluated in a phase I clinical trial (Table 8).

Datopotamab deruxtecan (Dato-DXd) consists of a monoclonal antibody targeting Trop-2, DXd, a topoisomerase I inhibitor, and a cleavable tetrapeptide junction [260]. Dato-DXd has shown favorable results in mTNBC patients [261]. DS-8201a (T-DXd), is a HER2-targeted ADC, composed of an anti-HER2 antibody and a derivative from the topoisomerase I inhibitor DX-8951 (DXd) [262]. Phase II/III clinical trials exhibited that DS-8201a displayed reliable tumor inhibitory activity in HER2^+^ metastatic BC patients and was approved for treating metastatic HER2^+^ BC [263, 264]. Intriguingly, DS-8201a also presented a meaningful antitumor activity in tumors with low HER2 expression [265, 266]. In another clinical trial, DS-8201a presented good antitumor activity in patients with low HER2 expression of BC [267]. The recently published DESTINY-Breast 04 trial indicated that in patients with advanced low HER2 expression BC, DS-8201a prolonged patient PFS and OS versus chemotherapy. Patients in the BC hormone receptor-negative subgroup had a 5.6 months mPFS and a 54% reduction in disease progression or death risk in the DS-8201a group, compared to the chemotherapy group. The mOS was extended to 9.9 months and the death risk decreased to 52% [268].

Nectin-4, a type I transmembrane cell adhesion molecule, is involved in the formation and maintenance of adherens junctions in cooperation with cadherins. Rabet et al. showed that nectin-4 is a cell surface biomarker frequently overexpressed in TNBC. They developed anti-nectin 4 ADC, N41mab-vcMMAE, which induced a complete and durable response in vitro and in vivo on nectin-4-positive samples [269]. In addition, Guo et al. developed a well-designed ADC, IC1-MMAE, as a potent targeted therapeutic agent for treating refractory TNBC in vivo. They provided experimental evidence of using ICAM1 as an effective ADC target for TNBC [270].

Based on the excellent efficacy of Trop-2-based ADCs in TNBC, more ADCs are in clinical trials, and we expect to see more benefits for patients. Moreover, the extension of DS-8201a therapy into the field of HER2 low expression is innovative and may cause new therapeutic options for breast cancer.

## Targeting regulated cell death (RCD)

In recent years, regulated cell death is associated with cancer progression and treatment, and ferroptosis is an iron-dependent type of RCD, which is not dependent on caspase cascade reaction [271–273]. In addition, ferroptosis has accumulated lipid peroxidation products and lethal reactive oxygen species (ROS) [274]. Gan et al. proposed that a ferroptosis inducer, IR780-SPhF, could enable TNBC imaging and treatment by targeting mitochondria and that IR780-SPhF had a stronger anticancer effect than cyclophosphamide, suggesting that IR780-SPhF could hold promise for the treatment of TNBC patients [275]. Glutathione peroxidase 4 (GPX4), an antioxidant enzyme, acts as an inhibitor of ferroptosis. Ding et al. found that DMOCPTL is capable of promoting the ferroptosis of TNBC cells by inducing GPX4 ubiquitination [276]. Prevention of GPX4-stimulated ferroptosis and increased sensitivity of TNBC cells in response to gefitinib [276]. The small-molecule compound erastin sensitizes TNBC cells to ferroptosis, but its application has been hampered by nephrotoxicity [277]. Yang et al. developed an exosome (erastin@FA-exo) that tagged folic acid (FA) and contained erastin. Erastin@FA-exo inhibits GPX4 expression and promotes ferroptosis in TNBC cells, and also inhibits TNBC cell proliferation more strongly than regular erastin [278]. This provides a novel therapeutic approach and direction for TNBC therapy. Tumor-associated macrophages (TAMs) are a critical element in the tumor microenvironment and are involved in tumor initiation and progression [279, 280]. IL-6 generated from TNBC cells stimulates TGF-β1 secretion by TAMs, which in turn allows hepatic leukemia factor (HLF) to trigger γ-glutamyl transferase 1 (GGT1) to promote ferroptosis resistance in TNBC cells, eventually causing the progression of TNBC [281]. In addition, iron-saturated Lf facilitated ferroptosis in TNBC cells via the production of ROS and enhanced the sensitivity of TNBC to radiotherapy [282]. Moreover, Zhang et al. indicated that MTHFD2 knockdown could induce ferroptosis in TNBC and inhibit TNBC progression, and may be a promising therapeutic target [283]. In the presence of ACSL3, mammary adipocytes protected TNBC cells from ferroptosis via oleic acid, which may offer new insights and targets for tumor therapy [284].

Interestingly, Zhimin Shao’s group revealed that there is high metabolic heterogeneity within TNBC, with the LAR-type being the ferroptosis-sensitive subtype, and AR-driven GPX4 being a critical molecule for mediating ferroptosis in the LAR subtype of TNBC. Moreover, GPX4 inhibitors not only suppressed the proliferation of LAR subtype cells, but also remodeled the tumor microenvironment [285]. Therefore, the combination of GPX4 inhibitors with ICIs may involve a prospective therapeutic approach for LAR subtype TNBC.

## New models for triple-negative breast cancer research

In recent years, the maturation of organoid technology has opened up a novel tool for tumor modeling. Organoids are in vitro cultured 3D tumor tissues that can more accurately reflect information associated with primary tumors and provide a more accurate tumor model for precision medicine [286–288]. Guillen et al. discovered that patient-derived xenografts (PDXs)-derived organoid (PDxO) could be utilized to screen promising therapeutic drugs such as birinapant, which exhibited powerful antitumor activity in the TNBC-organoid, and which has been validated in PDXs [289, 290]. Chemotherapy resistance is also a major barrier in the current treatment of TNBC patients, and inhibition of lysyl oxidase (LOX) in TNBC-organoid has been identified to enhance drug penetration, restrict FAK/Src signaling pathway, and overcome chemoresistance in TNBC [291]. In addition, liquid biopsy is crucial for precise cancer diagnosis and therapy [292, 293]. Salvador et al. predicted response to neoadjuvant chemotherapy at the time of TNBC diagnosis using immunosuppression-related biomarkers in blood samples and tumor biopsies [294]. The aforementioned emerging technologies are guiding the diagnosis and treating TNBC, and we expect to obtain reassuring strategies for TNBC treatment through these technologies in the future.

## Prospects

TNBC is characterized by a highly aggressive, largely heterogeneous, and highly malignant nature. At the same time, TNBC patients are susceptible to drug resistance and have a poor prognosis. Currently, there is a lack of valid targeted strategies for TNBC patients, and chemotherapy remains the main treatment method. With histological research and in-depth analysis of TNBC molecular typing, targeted therapy, and immunotherapy, as well as individualized therapy guided by TNBC molecular typing, light is being shed on the precision treatment for patients with TNBC [295].

The progression of TNBC and its malignant biological behaviors involve the aberrant activation of multiple signaling pathways. Exploring these relevant signaling pathways could help us to better understand the pathogenesis of TNBC, develop molecules with more diagnostic value or molecular markers with precision prognostic value, and provide a theoretical basis for molecularly targeted tumor therapy. Various antitumor drugs that target abnormally activated signaling pathways have been developed and have achieved excellent results in the pre-clinical setting. It is expected that more and more targeted drugs will be used in clinical settings in the future, bringing hope to TNBC patients.

"Fudan typing" plays a pivotal role in promoting precise therapy for TNBC and guiding researchers towards a more profound comprehension of TNBC heterogeneity. This advancement enables tailoring treatment plans based on individual TNBC patient characteristics, facilitating precise clinical trials aimed at enhancing the prognosis of TNBC patients. Nonetheless, the existing therapeutic approaches for TNBC remain constrained. Enhancing the effectiveness of treatments for TNBC patients stands as a pressing concern and formidable challenge. For instance, there's a pressing need to comprehensively analyze viable clinical targets for TNBC patients, explore superior treatment strategies, and surmount instances of drug resistance.

## Data Availability

Not applicable.

## Abbreviations

TNBC: Triple-negative breast cancer
BC: Breast cancer
HER2: Human epidermal growth factor receptor-2
EGFR: Epidermal growth factor receptor
TROP-2: Trophoblast cell-surface antigen 2
FDA: Food and drug administration
BL1: Basal-like 1
BL2: Basal-like 2
MSL: Mesenchymal stem-like
IM: Immunomodulatory
LAR: Luminal androgen receptor
IHC: Immunohistochemistry
PARP: Poly (ADP-ribose) polymerase
OS: Overall survival
iDFS: Invasive disease-free survival
DDFS: Distant disease-free survival
ORR: Objective response rate
pCR: Pathologic complete response
CBR: Clinical benefit rate
mTNBC: Metastatic TNBC
mAb: Monoclonal antibody
DLL4: Delta-like ligand 4
CAR-T: Chimeric antigen receptor-T
NK: Natural killer
JAK: Janus kinase
TGF: Transforming growth factor
ADC: Antibody–drug conjugate
SG: Sacituzumab govitecan
PFS: Progression-free survival
HDAC: Histone deacetylase
ICIs: Immune checkpoint inhibitors
PD-1: Programmed death receptor-1
PD-L1: Programmed death ligand-1
RCD: Regulated cell death
ROS: Reactive oxygen species
GPX4: Glutathione peroxidase 4
FA: Folic acid
TAMs: Tumor-associated macrophages
HLF: Hepatic leukemia factor
GGT1: γ-Glutamyl transferase 1
PDXs: Patient-derived xenografts
LOX: Lysyl oxidase
